# The Heart Protection Effect of Alcalase Potato Protein Hydrolysate Is through IGF1R-PI3K-Akt Compensatory Reactivation in Aging Rats on High Fat Diets

**DOI:** 10.3390/ijms160510158

**Published:** 2015-05-05

**Authors:** Wei-Syun Hu, Wei-Jen Ting, Wen-Dee Chiang, Peiying Pai, Yu-Lan Yeh, Chung-Ho Chang, Wan-Teng Lin, Chih-Yang Huang

**Affiliations:** 1 PhD Program for Aging, China Medical University, Taichung 40402, Taiwan; E-Mail: duodenum222000@yahoo.com.tw; 2 Graduate Institute of Basic Medical Science, China Medical University, Taichung 40402, Taiwan; E-Mail: wei-jen.ting@outlook.com; 3 Department of Food Science, College of Agriculture, Tunghai University, Taichung 40704, Taiwan; E-Mail: wdc@thu.edu.tw; 4 Division of Cardiology, China Medical University Hospital, Taichung 40402, Taiwan; E-Mail: paidoctor1@gmail.com; 5 Department of Pathology, Changhua Christian Hospital, Changhua 50006, Taiwan; E-Mail: 1867@cch.org.tw; 6 Department of Medical Technology, Jen-Teh Junior College of Medicine, Nursing and Management, Miaoli 35664, Taiwan; 7 Institute of Cellular and System Medicine, National Health Research Institutes, Zhunan Town 35053, Taiwan; E-Mail: changch@nhri.org.tw; 8 Department of Hospitality Management, College of Agriculture, Tunghai University, Taichung 40704, Taiwan; 9 Graduate Institute of Chinese Medical Science, China Medical University, Taichung 40402, Taiwan; 10 Department of Health and Nutrition Biotechnology, Asia University, Taichung 41354, Taiwan

**Keywords:** hyperlipidemia, alcalase potato protein hydrolysate, aging

## Abstract

The prevalence of obesity is high in older adults. Alcalase potato protein hydrolysate (APPH), a nutraceutical food, might have greater benefits and be more economical than hypolipidemic drugs. In this study, serum lipid profiles and heart protective effects were evaluated in high fat diet (HFD) induced hyperlipidemia in aging rats treated with APPH (15, 45 and 75 mg/kg/day) and probucol (500 mg/kg/day). APPH treatments reduced serum triacylglycerol (TG), total cholesterol (TC), and low density lipoprotein (LDL) levels to the normal levels expressed in the control group. Additionally, the IGF1R-PI3K-Akt survival pathway was reactivated, and Fas-FADD (Fas-associated death domain) induced apoptosis was inhibited by APPH treatments (15 and 45 mg/kg/day) in HFD aging rat hearts. APPH (75 mg/kg/day) rather than probucol (500 mg/kg/day) treatment could reduce serum lipids without affecting HDL expression. The heart protective effect of APPH in aging rats with hyperlipidemia was through lowering serum lipids and enhancing the activation of the compensatory IGF1R-PI3K-Akt survival pathway.

## 1. Introduction

Human metabolism decreases with increasing age, indicating that the risks of obesity and metabolic syndrome increase with age. In fact, more than 34.9% of adults were obese (obesity defined as BMI > 30) in 2011 and 2012, and the prevalence rates of obesity were 39.5% in middle-aged adults and 35.4% in older adults [1]. Obesity can increase the burden on the heart, with deteriorating coronary circulation and atherogenesis [2]. Moderate exercise can reduce weight and decrease the loading on the heart; however, the requirement of exercise therapy for the elderly remains controversial; in particular, obesity and sarcopenia in the elderly simply render them unable to exercise.

Thus, the intake nutraceuticals foods with cholesterol-lowering functions could help the elderly to achieve health relatively easily [3]. In fact, these potentially nutraceutical foods include proteins, protein hydrolysates, and peptides sourced from plants, such as soybean [4]. Soy peptides have been reported to decrease the risk of cardiovascular disease [5,6,7]. Not only soy peptides but also potato protein hydrolysate (PPH) were discovered to have antioxidative activity and exert protective effects against ethanol-induced gastric mucosal damage [8]. Alcalase was reported to hydrolyze soy protein into antioxidative hydrolysates [9,10]. In this research, alcalase potato protein hydrolysate (APPH) was used, and elderly rats were treated with a high-fat diet to evaluate the cardiac protective function of APPH.

Several studies have shown that Fas (CD95) is associated with obesity and increases the risk of heart disease [11,12]. Our previous studies have indicated that increased expression of Fas will lead to increased myocardial apoptosis downstream of release of caspase-8 through Fas-associated death domain (FADD) [13]. An appropriate treatment must be able to exert protective effects on the heart through the reduction of Fas expression.

In addition, the IGF1R-PI3K-Akt signaling pathway was also confirmed in our previous studies to increase the cardiac cell survival rate in heart disease animal models [14]. Furthermore, our previous research revealed that the IGF1R-PI3K-Akt survival pathway was more compensatorily expressed in young rats fed high-fat diets than in elder rats [15]. There are many cardio-protective drugs, such as resveratrol, that could also exert protective effects on the heart via this pathway [16]. Recently, probucol was reported as a lipid-lowering drug and with antioxidant properties of HDL [17,18,19]. In this work, probucol was also employed as the positive control. The results of this experiment showed that the heart-protective effects of APPH were highly dependent on IGF1R-PI3K-Akt pathway activation and Fas apoptotic pathway reduction in an animal model of aging with a high-fat diet.

## 2. Results and Discussion

Several studies have shown that Fas (CD95) is associated with obesity and increases the risk of heart disease [11,12]. Our previous studies have indicated that increased expression of Fas will lead to increased myocardial apoptosis downstream of release of caspase-8 through FADD [13]. An appropriate treatment must be able to exert protective effects on the heart through the reduction of Fas expression. The serum lipid profile analysis results showed high expression levels not only of TG, TC, and LDL-C but also of HDL-C and GLU in the aging rats fed a high-fat diet (Table 1). After four weeks of APPH treatments, the TG, TC, and LDL-C expression levels were reduced, and the same result was observed following the dose increase of APPH from 15 to 75 mg/kg/day. Similarly, after four weeks of probucol (500 mg/kg/day), the treatments also reduced the TG, TC, and LDL-C expression levels. In particular, blood glucose was only reduced after four weeks of APPH (75 mg/kg/day) treatments and not with probucol. After four weeks of experiments, BUN and CRE expression showed no differences between the aging control group and the indicated treatment groups. Probucol has been reported to be an ABCA1 inhibitor, and it can be used to increase the rate of LDL catabolism [17,18]. In these experiments, four weeks of probucol treatment successfully reduced the TG, TC and LDL levels in the serum of aging rats fed a high-fat diet (Table 1).

**Table 1:** Serum biochemical analysis.

Groups	Control	HFD	APPH (15 mg)	APPH (45 mg)	APPH (75 mg)	Probucol (500 mg)
			HFD	HFD	HFD	HFD
BUN (mg/dL)	14.4 ± 3.1	15.5 ± 1.7	13.6 ± 2.1	12.5 ± 2.6	12.6 ± 2.6	11.9 ± 1.2
CRE (mg/dL)	0.4 ± 0.1	0.5 ± 0.2	0.6 ± 0.1	0.5 ± 0.1	0.4 ± 0.1	0.5 ± 0.1
TC (mg/dL)	74.0 ± 20.7 ^a^	182.7 ± 43.4	121.3 ± 23.9 ^a^	96 ± 21.5 ^a^	89.7 ± 12.9 ^a^	84.3 ± 19.5 ^a^
TG (mg/dL)	122.3 ± 13.4 ^a^	152.7 ± 17.6	92.3 ± 12.1 ^a^	83.0 ± 19.7 ^a^	67.0 ± 16.6 ^a^	63.3 ± 11.0 ^a^
HDL-C (mg/dL)	27.0 ± 10.4	35.2 ± 8.8	34.7 ± 9.1	31.7 ± 7.8	27.6 ± 7.9 ^a^	23.3 ± 4.2 ^a^
LDL-C (mg/dL)	107.3 ± 12.9 ^a^	227.0 ± 14.6	185.5 ± 12.6 ^a^	92.3 ± 3.1 ^a^	81.0 ± 3.0 ^a^	74.3 ± 12.7 ^a^
GLU (mg/dL)	107.0 ± 35.2 ^a^	183.3 ± 39.6	173.7 ± 34.0	135.0 ± 20.1 ^a^	99.3 ± 13.3 ^a^	127.0 ± 32.0 ^a^

^a^ The *p*-value <0.001 compared with HFD group. APPH: alcalase potato protein hydrolysate; BUN: blood urea nitrogen; CRE: serum creatinine; TC: total cholesterol; TG: triacylglycerol; HDL-C: high-density lipoprotein cholesterol; LDL-C: low-density lipoprotein cholesterol; GLU: blood glucose.

Unfortunately, HDL was also slightly reduced by probucol treatment, which is the main reason that probucol clinical trials were stopped [19,20]. Although APPH (75 mg/kg/day) treatments could reduce the TG, TC and LDL levels in the serum of aging rats fed a high-fat diet, HDL was also reduced, and there were no significant differences between the aging control and APPH (75 mg/kg/day) treatment groups. Furthermore, blood glucose was reduced in the APPH (75 mg/kg/day) treatment group and was similar to the aging control group. These results suggest that APPH might lower serum lipids with fewer side effects than probucol. Spielmann discovered with hypolipidemic effects of potato protein in pigs [21]. But in our daily food preparation, the heating process results in loss of functional properties of the potato proteins [22]. In this work, alcalase hydrolysis of potato proteins with limited peptide cleavage under controlled conditions has been employed to produce hypolipidemic active peptides from potato protein [23]. In our pervious analysis using ESI-MS/MS spectrum obtained from reverse-phase high-performance liquid chromatography analysis indicated that papatin might be one of the hypolipidemic effects peptides of APPH [24].

**Figure 1:**
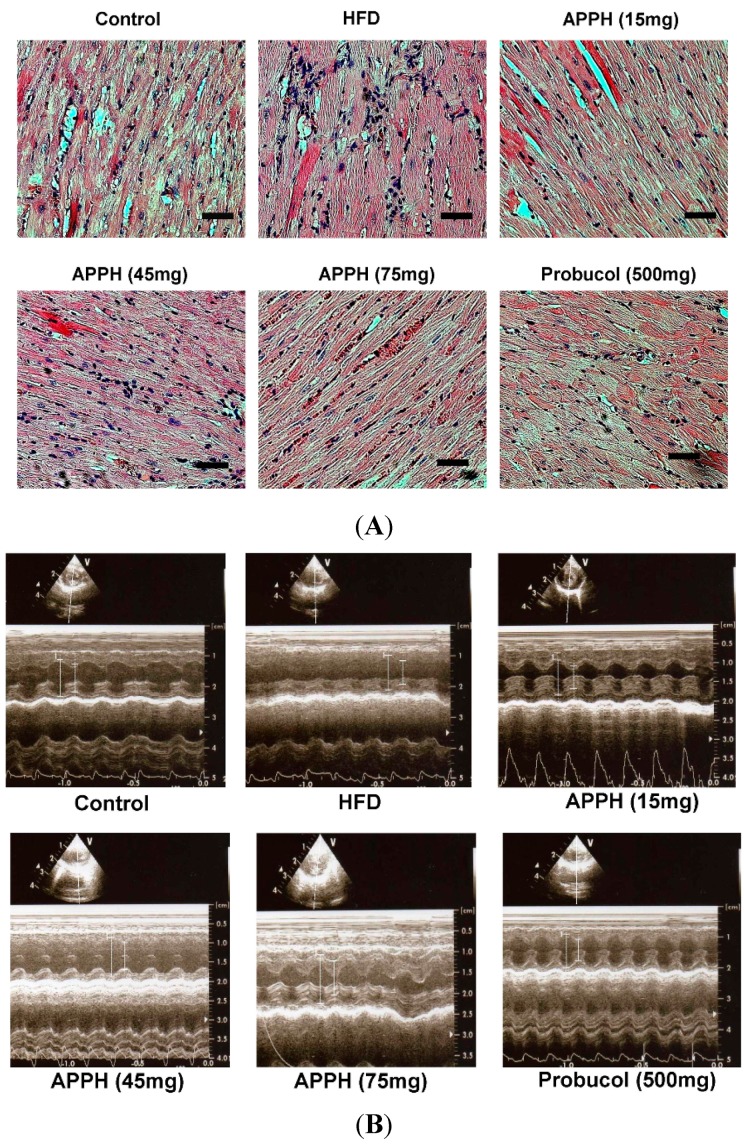
Asessments of cardiac features. (**A**) Hematoxylin and eosin (H & E) staining of rat hearts. Cell nuclei are stained in blue, and other intracellular or extracellular proteins are stained in pink. All heart sections were obtained from the ventricular septum of each rat (Bar length = 50 μm); (**B**) Echocardiography images of each group. The heart functions of each group were compared by the left ventricular systolic and diastolic distances (cm).

Hematoxylin and eosin (H & E) staining of heart tissue slides identified the cardiomyocytes. The histology of normality is characterized by a parallel alignment of myocardial fibers, and histology of myocardial disarray is characterized by a more complex texture and disorganization of myocardial cells [25]. Myocardial disarray was presented in both the aging group and the high-fat diet and aging group hearts (Figure 1). After four weeks of APPH (15 mg/kg/day) treatment, the myocardial disarray was slightly improved. With APPH treatments (45 and 75 mg/kg/day) and probucol (500 mg/kg/day) treatments, the disarrayed cardiomyocytes were significantly improved and arranged more closely.

After four weeks of experiments, cardiac echocardiography was applied to the rats for heart ejection fraction (EF) and fraction shortening (FS). The length measurements of left ventricular internal end-diastolic dimensions (LVIDd) of each group are shown as long bars, and the left ventricular internal end-systolic dimensions (LVIDs) of each group are shown as the short bars (Figure 1). The heart function of the aging control group, as shown by the EF, was 54.0% ± 5.2%, and the FS was 24.9% ± 3.0% (Table 2). In the high-fat diet aging group, the EF was 54.0% ± 4.7%, and the FS was 24.9% ± 2.7%. The EF in the groups given a high-fat diet combined with APPH treatment increased to 62.2% ± 4.6% in the APPH (15 mg/kg/day) treatment group, to 67.5% ± 5.6% in the APPH (45 mg/kg/day) treatment group and to 66.4% ± 3.2% in the APPH (15 mg/kg/day) treatment group. Similarly, the FS with the high fat diet combined with APPH treatments increased to 31.8% ± 2.9% in the APPH (15 mg/kg/day) treatment group, to 33.7% ± 4.1% in the APPH (45 mg/kg/day) treatment group and to 33.0% ± 2.2% in the APPH (75 mg/kg/day) treatment group. In addition, the EF increased to 69.4% ± 2.5% and the FS increased to 35.2% ± 1.8% in the high fat diet combined with probucol treatment group.

**Table 2:** Echocardiography.

Groups	Control	HFD	APPH (15 mg)	APPH (45 mg)	APPH (75 mg)	Probucol (500 mg)
			HFD	HFD	HFD	HFD
BW (g)	465.7 ± 63.9 ^a^	700.3 ± 88.6	690.3 ± 67.5	638.7 ± 71.6	607.2 ± 70.2 ^a^	688.2 ± 52.1
EF (%)	54.0 ± 5.2	54.0 ± 4.7	62.2 ± 4.6	67.5 ± 5.6 ^a^	66.4 ± 3.2 ^a^	69.4 ± 2.5 ^a^
%FS (%)	24.9 ± 3.0	24.9 ± 2.7	31.8 ± 2.9 ^a^	33.7 ± 4.1 ^a^	33.0 ± 2.2 ^a^	35.2 ± 1.8 ^a^

^a^ The *p*-value <0.001 compared with HFD group. BW: body weight; EF: ejection fraction; FS: fraction shortening.

**Figure 2:**
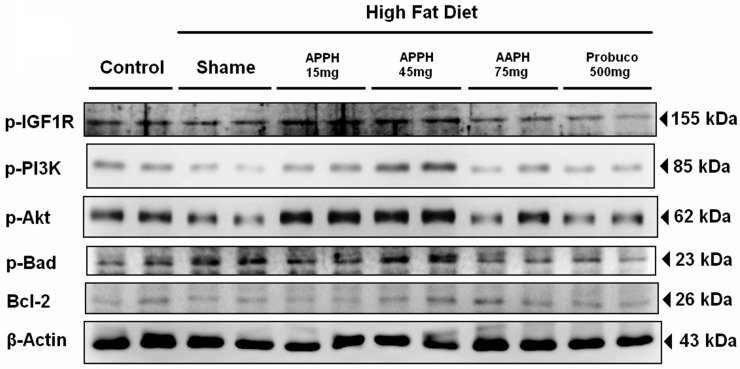

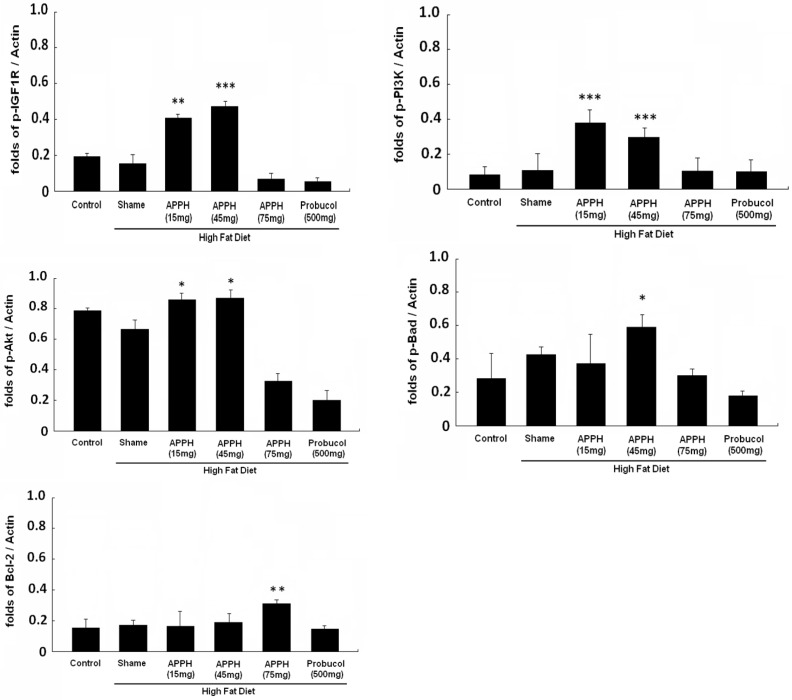
IGF1R-regulated survival pathway signaling analysis. All protein samples from each rat group were analyzed by Western blotting (*n* = 3). The protein expression folds were normalized with β-actin. * *p* < 0.05, ** *p* < 0.01, *** *p* < 0.001 compared with the HFD (high-fat diet) group.

The survival signaling pathway protein expression in the heart tissue from each group after four weeks of the indicated treatments was analyzed by Western blotting assay. The cell survival protein levels of p-IGF1R, p-PI3K, p-Akt and downstream p-Bad were not strong in either the aging or high fat diet aging group hearts (Figure 2). However, the levels of p-IGF1R, p-PI3K, p-Akt and p-Bad expression were increased in the 15 and 45 mg/kg/day APPH treatment groups. However, 75 mg/kg/day APPH treatment and 500 mg/kg/day probucol treatment did not enhance the expressions of p-IGF1R, p-PI3K, p-Akt or p-Bad. In our previous studies, we discovered that the IGF1R-PI3K-Akt survival pathway plays an important role in heart protection, particularly in hyperlipidemia induced by a high-fat diet [26,27]. Unfortunately, the IGF1R-PI3K-Akt survival pathway could be more easily compensatorily reactivated through exercise training in young rats, and the originally compensative expression was almost lost [15]. The downstream molecule p-Bad, an anti-apoptosis protein, was upregulated; its phosphorylation expression by p-Akt and covered it with 14-3-3 [28,29]. In this work, APPH (15 and 45 mg/kg/day) treatments reactivated the IGF1R-PI3K-Akt signaling pathway and downstream p-Bad expression in the hearts of aging rats fed a high-fat diet (Figure 2). Bcl-2 is also an anti-apoptotic protein but not a downstream protein of the IGF1R-PI3K-Akt signaling pathway. The expression of Bcl-2 was also evaluated and showed no significant differences among the APPH treatment groups. Thus, the IGF1R-PI3K-Akt signaling pathway with APPH 15 and 45 mg/kg/day might constitute the original physiological phenomenon that was compensatorily reactivated by the serum lipid reducing effects of APPH [14,15,30]. When the TG, TC and LDL levels were reduced by APPH (75 mg/kg/day) or probucol (500 mg/kg/day), and the expression levels were close to those in the control group, activation of the compensative IGF1R-PI3K-Akt signaling pathway might not be necessary (Table 1).

**Figure 3:**
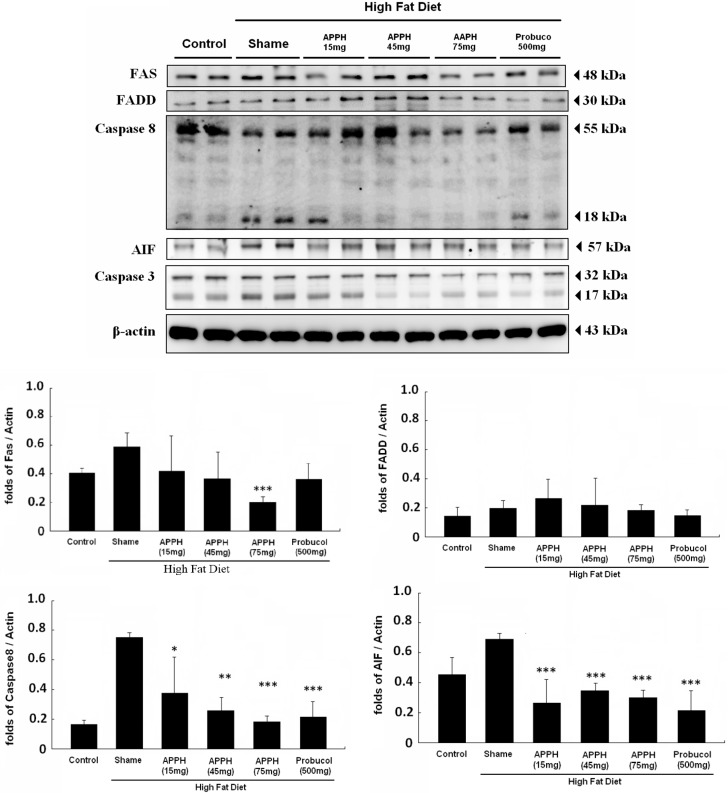

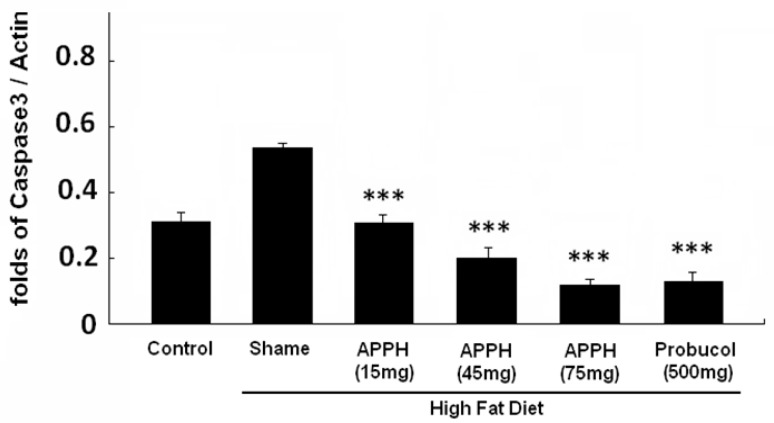
Fas-induced apoptosis pathway signaling analysis. All protein samples from each rat group were analyzed by Western blotting (*n* = 3). The protein expression folds were normalized with β-actin. * *p* < 0.05, ** *p* < 0.01, *** *p* < 0.001 compared with the HFD (high-fat diet) group.

In our previous research discovered, HFD treatment will increase FAS expression and cause FAS-induced cardiac apoptosis [31]. The expression of Fas-induced apoptosis signaling pathway proteins in the heart tissues of each group after four weeks of the indicated treatments were analyzed by Western blotting assay (Figure 3). The proteins of Fas, FADD, caspase-8 and downstream caspase-3 were expressed at higher levels in both the aging and high-fat diet group hearts. After four weeks 15 and 45 mg/kg/day APPH treatments did not reduced the Fas, FADD, caspase-8 or caspase-3 protein levels significantly. Only the 75 mg/kg/day APPH treatment and 500 mg/kg/day probucol treatments could inhibited the expression of Fas-induced apoptosis signaling pathway proteins, such as Fas, FADD, caspase-8 and caspase-3. Some protein hydrolysates have been discovered that can modulate the expression of proinflammatory cytokines [32]. Here, we also evaluated Fas expression in the hearts of aging rats treated with APPH and fed a high-fat diet. As the results showed, the Fas protein level was reduced with APPH 75 mg/kg/day and probucol 500 mg/kg/day treatments (Figure 3). Not only Fas but also its downstream counterparts FADD, caspase-8 and caspase-3 were reduced by APPH and probucol treatments.

**Figure 4:**
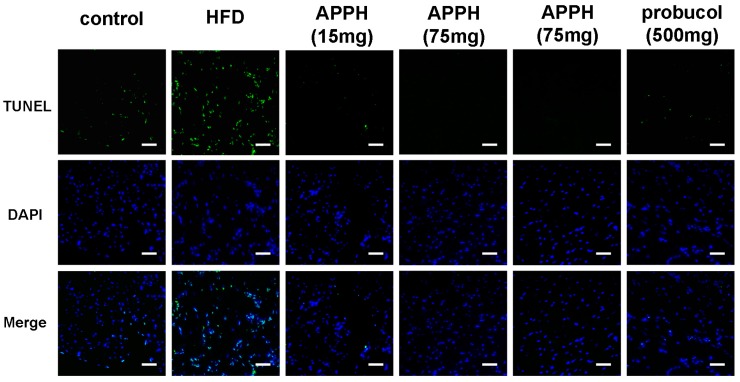

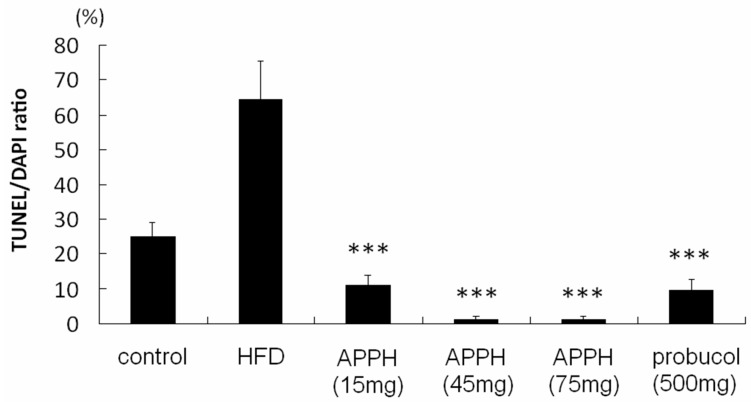
DAPI and TUNEL staining analysis. Cell nuclei were stained blue using DAPI on the heart biopsy slides, and specific DNA fragments in apoptotic cell nuclei were stained green using TUNEL (Bar length = 100 μm). The percentage of apoptosis cells is presented as the TUNEL/DAPI ratio. *** *p* < 0.001 compared with the HFD (high-fat diet) group.

The HFD induced cardiac apoptosis might not only induced from Fas-FADD expression, but also can be induced mediated by Il-6 and causes down-regulation of PPARs [33]. HFD treatment will also decrease the cardiac leptin receptor, induced lopotoxicity, cardiac oxidative damage, inflammation, and cell death in cardiomyocyte [34,35,36]. On the slide images, the cell nuclei were indicated by DAPI staining (blue), and the specific DNA fragments caused by caspase cleavage during apoptosis were stained by TUNEL (green) (Figure 4). In the aging control group, a few apoptotic cells were present. However, a large number of apoptotic cardiomyocytes appeared in the high fat diet group hearts. After APPH treatment, apoptotic cardiomyocytes were reduced significantly in the 15, 45 and 75 mg/kg/day groups. The apoptotic cardiomyocyte numbers were also reduced in the probucol treatment group hearts. Furthermore, the DAPI and TUNEL staining assay also proved that APPH and probucol treatments reduced apoptotic cardiomyocytes (Figure 4). Interestingly, although both APPH and probucol could reduce the serum lipids and Fas-FADD signaling pathway proteins, the mechanism by which APPH affected Fas-FADD signaling in hyperlipidemic rat hearts remained unclear.

## 3. Experimental Section

### 3.1. Chemicals

Probucol was purchased from Sigma-Aldrich (St. Louis, MO, USA), and potato protein was purchased from Han-Sient Trading Co., Ltd. (Taipei, Taiwan). Alcalase was purchased from Novo Nordisk A/S (Copenhagen, Denmark). The 2.5% potato protein water solution was combined with alcalase (1% weight of enzyme/w of potato protein) at the temperature and pH suggested by the supplier’s directions for 6 h, when the degree of hydrolysis (DH) reached 19%. The DH measurement of the potato protein hydrolysate (PPH) was modified by the ortho-phthaldialdehyde (OPA) method [16]. In brief, 50 μL of the PPH was combined with OPA aqua-solution (0.5 mL of 100 mM sodium tetraborate, 50 μL of 20% SDS, 0.8 mg of OPA, 2 μL of β-mercaptoethanol) for a total of 1 mL and was incubated at room temperature for 2 min; then, the absorbance was measured at a 340 nm wavelength using ultraviolet-visible spectrophotometry (Shimazu, UVmini-1240). The APPH product used in this work had been produced at one-time.

### 3.2. Animals

The animal use experimental protocol was approved by the Institutional Animal Care and Use Committee (IACUC) of China Medical University (No. 101-263-B). All of the Sprague-Dawley (SD) rats used were 5 months of age and were purchased from BioLASCO Taiwan Co., Ltd. (Taipei, Taiwan). The SD rats were raised in an animal center at China Medical University until they were 22 months of age; then, they were divided into 6 groups (*n* = 6 each): 5 groups were fed a high-fat diet, and 1 group was fed a standard diet until the rats were 24 month of age. When the rats were 23 months of age, they were given the indicated treatments as follows. The control group rats were fed normal water and standard diets (Laboratory Rodent Diet 5001 purchased from LabDiet, St. Louis, MO, USA). The high-fat diet formula consisted of cholesterol (Sigma) mixed until 3% (in *w*/*w*) 58Y1 (Diet Induced Obesity Rodent Purified Diet with 60% Energy From Fat); 58Y1 was purchased from LabDiet. The high-fat diet group rats were fed normal water and a high-fat diet. The APPH treatment group rats were not only fed normal water and high-fat diet, but they were also individually treated with low-dose APPH (15 mg/kg/day), medium-dose APPH (45 mg/kg/day) and high-dose APPH (75 mg/kg/day). The probucol treatment group rats were fed normal water, a high-fat diet and probucol (500 mg/kg/day).

### 3.3. Echocardiography

Echocardiographic examinations were performed using Vivid i ultrasound system (GE Healthcare, Milwaukee, WS, USA) with a 10 MHz transducer (GE 10S-RS) for acquisitions, along the parasternal long-axis and short-axis, of the views of left ventricle obtained at the level of the papillary muscles for two-dimensional mode images (B-mode). These B-mode views were used in determining the optimal position perpendicular to the ventricular septum and posterior wall of left ventricle for M-mode cursor. M-mode tracings recorded the anterior and posterior Left ventricular (LV) walls thicknesses, left ventricular internal end-diastolic dimensions (LVIDd), left ventricular internal end-systolic dimensions (LVIDs), end diastolic volume (EDV), end systolic volume (ESV), ejection fraction (EF) and fractional shortening (FS). These parameters were measured, with modified leading edge methods of the American Society of Echocardiography, from at least three consecutive cardiac cycles on the M-mode tracings in rat hearts (*n* = 6). EF% was calculated by: (EDV − ESV)/EDV × 100. FS% was calculated according to the following equation: FS% = [(LVIDd − LVIDs)/LVIDd] × 100.

### 3.4. Hematoxylin and Eosin Staining

The hearts of rats in each group were soaked in formalin, dehydrated through graded alcohols, and embedded in paraffin wax. Subsequently, 2 μm-thick paraffin sections were cut from these paraffin-embedded tissue blocks. The tissue sections were deparaffinized by immersing them in xylene and were rehydrated. All slices were stained with hematoxylin and eosin (H & E) and then rinsed with water. Each slide was dehydrated through graded alcohols. Finally, the slides were soaked in xylene twice. Photomicrographs were obtained using Zeiss Axiophot microscopes in a 200× view field.

### 3.5. DAPI and TUNEL Staining

For the terminal deoxynucleotidyl transferase dUTP-mediated nick-end labeling (TUNEL) assay, all of the heart tissue slides were incubated with proteinase K (2 μg/mL) for 15 min and then were washed in PBS twice. Next, they were incubated with 0.1% sodium citrate solution (with 0.1% Triton X-100), soaked in blocking buffer and washed twice with PBS. The terminal deoxynucleotidyl transferase and fluorescein isothiocyanate-dUTP apoptosis detection kit (Roche Applied Science, Indianapolis, IN, USA) was used for 60 min at 37 °C. TUNEL-positive nuclei (fragmented DNA) were fluoresced by bright green light at 460 nm. All cell nuclei of the tissue slides were fluoresced by blue light at 454 nm after 5 min of incubation with 4,6-diamidino-2-phenylindole (DAPI) solution (0.1 μg/mL DAPI in PBS). Photomicrographs were obtained using a Zeiss Axiophot microscope in a 400× view field.

### 3.6. Blood Biochemical Analysis

In this study, all plasma samples were collected and measured by China Medical University Hospital, and the following parameters were analyzed: CRE (serum creatinine), TG (triacylglycerol), TC (total cholesterol), LDL-C (low-density lipoprotein cholesterol), HDL-C (high-density lipoprotein cholesterol), GLU (blood glucose) and BUN (blood urea nitrogen). CRE analysis was used for monitoring the function of kidney and BUN analysis was used for monitoring the function of liver. TG, TC, LDL-C and HDL-C analysis presented the lipid profile in serum. The GLU presented the blood glucose level.

### 3.7. Tissue Protein Extraction

Heart tissue extracts of 6 rats from each group were obtained by homogenizing the tissue in lysis buffer (0.05 M Tris-HCl, pH 7.4, 0.15 M·NaCl, 0.25% deoxycholic acid, 1% NP-40, 1 mM EDTA) at a ratio of 100 mg tissue/1 mL buffer. The homogenates were placed on ice and then were centrifuged at 13,000 rpm for 40 min. The supernatants were collected and stored at −80 °C for further experiments.

### 3.8. Western Blot Assay

The protein concentrations of the heart tissue extracts were determined by the Lowry protein assay. Protein samples were separated by 12% SDS polyacrylamide gel electrophoresis (SDS-PAGE), with a constant voltage of 75 V for 120 min. Proteins were then transferred to Hybond-C membranes (GE Healthcare UK Ltd., Buckinghamshire, UK) using 50 V for 3 h. PVDF membranes were incubated in 3% bovine serum albumin (BSA) in TBS buffer. Primary antibodies, including p-IGF1R (ab-39398, Abcam, Cambridge, UK), p-PI3K (SC-12929, Santa Cruz Biotechnology, Santa Cruz, CA, USA), p-Akt (#9271, Cell Signaling, Baltimore, MD, USA), p-Bad (SC-7999, Santa Cruz Biotechnology), Bcl-2 (SC-7382, Santa Cruz Biotechnology), β-actin (SC-47778, Santa Cruz Biotechnology), Fas (SC-7886, Santa Cruz Biotechnology), FADD (SC-6035, Santa Cruz Biotechnology), caspase-8 (SC-6134, Santa Cruz Biotechnology), AIF (SC-19128, Santa Cruz Biotechnology), and caspase-3 (SC-7148, Santa Cruz Biotechnology), were added to the membranes to recognize the fitted proteins. Finally, horseradish peroxidase-labeled antibodies were used, and images were obtained with Fujifilm LAS-3000 (GE Healthcare UK Ltd.).

### 3.9. Statistical Analysis

The results shown are the means ± SDs of three independent experiments. Statistical analysis was performed by one-way analysis of variance. For paired samples, Student’s *t* test was applied.

## 4. Conclusions

Both APPH and probucol treatments could reduce the serum lipids, such as TC, TG, and LDL. However, APPH treatment did not decrease HDL levels compared to probucol treatment in high-fat diet aging rats. Furthermore, APPH could reactivate the compensatory IGF1R-PI3K-Akt survival pathway. In this work, APPH treatments indeed provided physiologic protection to the hearts of aging rats with hyperlipidemia induced by a high-fat diet.
